# Protective Effects of Chromium Picolinate Against Diabetic-Induced Renal Dysfunction and Renal Fibrosis in Streptozotocin-Induced Diabetic Rats

**DOI:** 10.3390/biom10030398

**Published:** 2020-03-04

**Authors:** Shan Shan Qi, Hong Xing Zheng, Hai Jiang, Li Ping Yuan, Le Chen Dong

**Affiliations:** 1 Vitamin D research institute, College of Biological Science and Engineering, Shaanxi University of Technology, Hanzhong 723000, Shaanxi, China; jianghai0318@163.com; 2 College of Biological Science and Engineering, Shaanxi University of Technology, Hanzhong 723000, Shaanxi, China; yuanliping@stu.snut.edu.cn (L.P.Y.); donglechen@stu.snut.edu.cn (L.C.D.)

**Keywords:** diabetic nephropathy, chromium picolinate, renal fibrosis, antioxidative stress

## Abstract

Diabetic nephropathy (DN) is one of the most important complications of diabetes, and the leading cause of end-stage renal disease (ESRD). While Chromium picolinate (CrPic) supplementation has been found to be effective in treating diabetes, its effects on diabetic-induced nephropathy have not been studied. Therefore, in this study, CrPic (1 mg kg^−1^ d^−1^) was administered to a DN rat model by oral gavage for eight weeks to investigate its effects. The results show that CrPic supplementation caused a decrease in levels of blood glucose, serum insulin, blood urea nitrogen (BUN), serum creatinine, and urinary albumin in DN rats. It also reversed renal pathological changes, including renal glomerular sclerosis and interstitial fibrosis. In addition, the oxidative defense system in the kidneys of DN rats was found to be improved; the biological activities of superoxide dismutase (SOD), catalase (CAT), and glutathione peroxidase (GPX) increased; and the content of malondialdehyde (MDA) lowered. Immunohistochemical results reveal that the expression levels of renal transforming growth factor-β1 (TGF-β1), Smad 2, and Smad 3 decreased significantly in the kidneys of rats in the CrPic-treated group. CrPic administration was thus found to ameliorate diabetic nephropathy in SD rats via an antioxidative stress mechanism, as well the ability to inhibit TGF-β1/Smad2/3 expression. This study suggests that CrPic could be a potential renal-protective nutrient against diabetic nephropathy.

## 1. Introduction

The global prevalence of diabetes is on a rapid rise due to changes in lifestyles and eating habits. According to a report by the International Diabetes Federation (IDF), there were about 451 million people (aged 18–99) with diabetes globally in 2017, which is estimated to increase to 693 million by 2045 [1]. Diabetes has, indeed, become a major threat to human health. 

Diabetic nephropathy (DN) is a severe complication of diabetes and the leading cause of death and end-stage renal disease (ESRD). About 30% of diabetic patients have kidney disease, proteinuria, and other severe symptoms, and eventually develop diabetic nephropathy; 53% of patients die from ESRD [2]. As a chronic disease that endangers human health, DN has become an important public health and social problem. The main clinical manifestations of DN are proteinuria, special renal morphology, and functional changes. The main pathological characteristics of this disease are excessive accumulation of mesangial cells and extracellular matrix (ECM), leading to thickening of the glomerular basement membrane. This further causes renal dysfunction, renal fibrosis, and glomerular sclerosis, eventually resulting in ESRD [3,4,5]. Thus, there is the urgent need to carry out research to prevent and control diabetic nephropathy, propose effective prevention strategies, and develop effective preventive measures (targeted drugs and nutritional supplements). 

Chromium picolinate (CrPic) is an essential trace element that has long been used to treat type 1, type 2, gestational, and steroid-induced diabetes. It is acknowledged to lower blood glucose levels, and enhance insulin function and sensitivity [6,7,8,9]. CrPic has also been known to have antioxidant properties and a beneficial effect on diabetes-induced atherosclerosis [10,11]. However, it is not reported to have a preventive effect on diabetes-induced DN, especially renal dysfunction and renal fibrosis. Thus, in this study, we created a DN rat model using streptozotocin (STZ) and administered CrPic to evaluate its therapeutic effects and possible antioxidant mechanism. This study will provide new theories and methods for the adjuvant treatment of DN, and provide a theoretical basis for the application of CrPic in controlling diabetes by diet.

## 2. Materials and Methods

### 2.1. Chemicals and Reagents

CrPic was purchased from Nutrition 21 (New York, USA); Streptozotocin (STZ) from Aladdin Reagent (Shanghai, China); ELISA kits and malondialdehyde (MDA), superoxide dismutase (SOD), catalase (CAT), and glutathione peroxidase (GPX) detection kits from Shanghai Enzyme-linked Biotechnology Institute (Shanghai, China); and immunohistochemistry antibodies and DAB from Bioss Biological Technology (Beijing, China).

### 2.2. Animals

SD rats (eight-week-old male) were purchased from Chendu Dashuo Experimental Animal Co., Ltd. (Chengdu, China) (licence no. SCXK (Chuan) 2015-030). The rats were housed in a standard animal room (5 or 6 per cage) with 22 ± 1 °C, 70% humidity, and 12 h light and dark cycles. They were fed an ad libitum food and water diet for three days prior to the experiment. Animal procedures and NIH care guidelines were adhered to, and all experimental animal protocols were approved by the Ethics Committee of Shaanxi University of Technology. 

### 2.3. Diabetic Nephropathy Induction and CrPic Administration

The rats were randomly distributed into two groups after seven days of acclimation: a control group (*n* = 8) and a DN group (*n* = 40). The rats in the DN group were intraperitoneally injected with streptozotocin (STZ) (45 mg kg^−1^, STZ was dissolved in citrate buffer, pH = 4.0), and the control rats were injected with citric acid buffer solution. Three days after the STZ injection, tests were conducted to assess morning non-fasting blood glucose and urinary protein levels. Rats with a non-fasting blood glucose level higher than 16.7 mmol/L and positive urine protein were considered to have diabetic nephropathy and were used in this study.

Thereafter, the rats were randomly distributed into four groups: (i) control group (CON; deionized water; oral gavage; *n* = 8); (ii) diabetic nephropathy group (DN; deionized water; oral gavage; n = 8); (iii) diabetic nephropathy rats administered with CrPic (DN-Cr; 1 mg kg^−1^ d^−1^; oral gavage; n = 8); and (iv) diabetic nephropathy rats administered with metformin as the positive control (DN-Met; 150 mg kg^−1^ d^−1^; oral gavage; *n* = 8). During the experimental period, random blood glucose, body weight, food, and water intake was measured every week. On the last day of administration, the rats were placed in metabolic cages and 24-hour urine was collected for detection of urinary albumin concentration. The rats were then sacrificed, blood samples collected from the heart and centrifuged at 3000 rpm for 10 min, and serum samples stored at −80°C for biochemical tests. Kidney tissues were collected for H&E, PAS, Masson’s trichrome, and immunohistochemical detection, and the kidney index was calculated using the following formula:

Kidney index (mg/g) = kidney weight (mg)/body weight (g)(1)

### 2.4. Renal Function Assessment

Urine albumin concentration was detected on a microplate reader (BioTek EL800, Winooski, Vermont, USA) using the ELISA kit according to the manufacturer’s instructions. An automatic biochemistry analyzer (Modular P800, Roche, Mannheim, Germany) was used to measure the levels of serum creatinine (Scr) and blood urea nitrogen (BUN).

### 2.5. Blood Glucose and Serum Insulin Measurement

At the end of the experiment, the rats were sacrificed with an overdose of isoflurane after anesthesia. Blood glucose was measured from the tail vein using a glucometer (Roche Diagnostics, Indianapolis, IN, USA). Blood was taken from the heart and centrifuged for 10 min at 4 °C to extract serum; an ELISA kit was used to detect serum insulin.

### 2.6. Renal MDA, SOD, CAT, and GPX Determinations 

Renal tissues were homogenized in cold PBS, and the homogenate was centrifuged at 4 °C at 900×g for 15 minutes; then the supernatant was collected. Malondialdehyde (MDA), superoxide dismutase (SOD), catalase (CAT), and glutathione peroxidase (GPX) levels in kidney homogenate were detected by using commercial assay kits (Shanghai Enzyme-linked Biotechnology Institute, Shanghai, China) according to the manufacturer’s instructions.

### 2.7. Histological Evaluation of Renal Pathological Changes 

Freshly dissected kidney tissues were fixed in 4% paraformaldehyde (PFA) for 24 hours. Thereafter, the tissues were dehydrated using graded ethanol (70%, 80%, 90%, 95%, 100%), made transparent with xylene, and embedded in paraffin. The samples were then sliced into 5-μm sheets using a RM2235 paraffin tissue slicer (Leica, Frankfurt, Germany). The tissue slices were stained with hematoxylin and eosin, and examined using an optical microscope (Leica Microsystems, Frankfurt, Germany). Renal histology was analyzed by a nephropathologist who was blinded to the treatment group. Renal pathological damage was scored according to a renal pathology scoring system. The scoring system has a total of five grades, which are defined according to the severity of kidney damage. For each rat, ten randomly selected fields of view (magnification: ×200) were examined under a microscope to assess kidney damage and average scores were calculated [12].

### 2.8. Masson’s Trichrome Staining for Evaluation of Renal Interstitial Fibrosis 

Five-micrometer kidney slides were stained with Masson’s trichrome (Bioss Biological Technology Company, Beijing, China) according to the procedure outlined in the staining kit. After the staining, 20 fields were randomly selected on each slice to evaluate interstitial fibrosis (magnification: ×200). The results were calculated by Image Pro Plus 5.0 analysis software (Media Cybernetics, Silver Spring, USA) as the ratio of Masson positive stained area to the total field area.

### 2.9. PAS Staining for Evaluation of Glomerulosclerosis

Kidney sections were stained with periodate-Schiff (PAS) (Bioss Biological Technology Company, Beijing, China) according to the procedure outlined in the staining kit. The semi-quantitative scoring system was used to evaluate the degree of glomerular sclerosis. For each PAS-stained section, 20 glomeruli were observed under magnification of ×200, and classified as 0 to 4 lesions as per the following: 0: glomerular no sclerosis; 1: glomerular sclerosis area ≤ 25%; 2: 25% < glomerular sclerosis area ≤50%; 3: 50% < glomerular sclerosis area ≤75%; 4: 75% < glomerular sclerosis area ≤100%. Glomerulosclerosis index (GSI) was calculated as: (1×N1 + 2×N2 + 3×N3 + 4×N4)/N×100 (N1, 2, 3, 4 are the number of glomeruli that scored 1, 2, 3, 4; N is the total number of glomeruli) [13].

### 2.10. Immunohistochemistry (IHC)

The kidney paraffin slices were baked at 60 °C for 1 h, dehydrated using graded ethanol (70%, 80%, 90%, 95%, 100%), repaired in citric acid solution (pH 6.0) at 90 °C for 1 h, washed with PBS-T 3 times, blocked in 3% H_2_0_2_ for 15 min and in goat serum for 10 min, and then incubated in TGF-β1, Smad 2, and Smad 3 primary antibody (Bioss Biological Technology Company, Beijing, China) solution (1:200) overnight at 4 °C. The slides were then rinsed with PBS-T, incubated in HRP-conjugated secondary antibody (Bioss Biological Technology Company, Beijing, China) (1:300) for 2 h at 37 °C, then washed with PBS-T. They were incubated in DAB solution (Bioss Biological Technology Company, Beijing, China) for 1.5 min, and then counterstained with hematoxylin [14]. Quantitative analysis of the percentage of positive staining regions TGF-β1, Smad 2, and Smad 3 were performed using a 5.0 analysis software under microscope.

### 2.11. Statistical Analysis

All the experimental data were expressed as mean ± SD. The significant differences from two groups were assessed by one-way analysis of variance (ANOVA) using SPSS 19.0. Values of *P* < 0.05 were considered as statistically significant.

## 3. Results

### 3.1. CrPic Increases Body Weight and Decreases Food Intake and Water Consumption in Diabetic Nephropathy Rats

**Figure 1:**
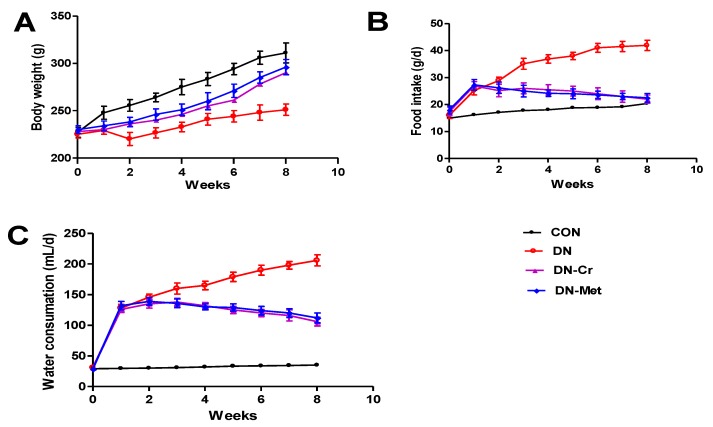
Body weight, food and water intake in each experimental group during the experiment. (**A**) The body weight in each experimental group; (**B**) The food intake in each experimental group; (**C**) The water intake in each experimental group. CON: control group; DN: diabetic nephropathy group; DN-Cr: diabetic nephropathy rats treated with CrPic (10 mg kg^−1^ d^−1^); DN-Met: diabetic nephropathy rats treated with metformin (150 mg kg^−1^ d^−1^).

As shown in Figure 1, the body weight of the DN group rats was significantly lower than that of the control group (*P* < 0.01). Food intake and water consumption of the DN group rats was higher than that of the control group (*P* < 0.01). After eight weeks of CrPic administration, the body weight of the rats in the CrPic-treated group increased, and their water and food intake decreased. There were significant differences between the DN-Cr and DN groups in body weight, and food or water intake by the end of experiment. This indicates that CrPic can reduce water and feed intake in diabetic nephropathy rats and prevent weight loss in diabetic rats.

### 3.2. CrPic Decreases Blood Glucose and Serum Insulin Levels in Diabetic Nephropathy Rats

**Figure 2:**
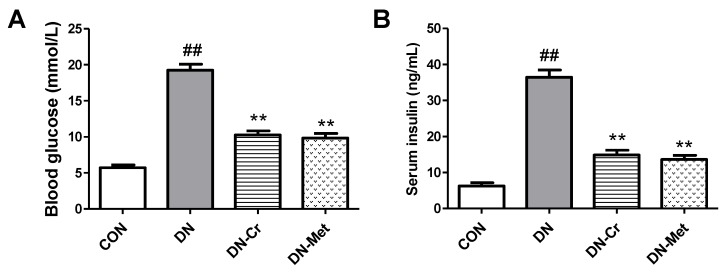
Blood glucose and serum insulin levels in each experimental group. CON. control group; (**A**) The Blood glucose levels in each experimental group; (**B**) The serum insulin levels in each experimental group; DN. diabetic nephropathy group; DN- Cr. diabetic nephropathy rats treated with CrPic (10 mg kg^−1^ d^−1^); DN-Met. diabetic nephropathy rats treated with metformin (150 mg kg^−1^ d^−1^). Compared to the control group, ## *P* < 0.01; Compared to the DN group, ** *P* < 0.01.

In order to examine the effects of CrPic on diabetes, blood glucose and serum insulin was detected. As Figure 2 indicates, the blood glucose and serum insulin levels in DN rats were higher than that of the control groups, and the differences were significant (blood glucose, DN vs. CON, *P* < 0.01; serum insulin, DN vs. CON, *P* < 0.01). After eight weeks of CrPic administration, the blood glucose and serum insulin levels decreased in the DN-Cr group, and there were significant differences in blood glucose and insulin between the CrPic- or metformin- treated group and the DN group (DN-Cr vs. DN, *P* < 0.01; DN-Met vs. DN, *P* < 0.01). This data suggests that CrPic exhibits anti-diabetic properties in STZ-induced diabetic rats. 

### 3.3. CrPic Decreases Kidney Index and Improves Renal Dysfunction in Diabetic Nephropathy Rats

**Figure 3:**
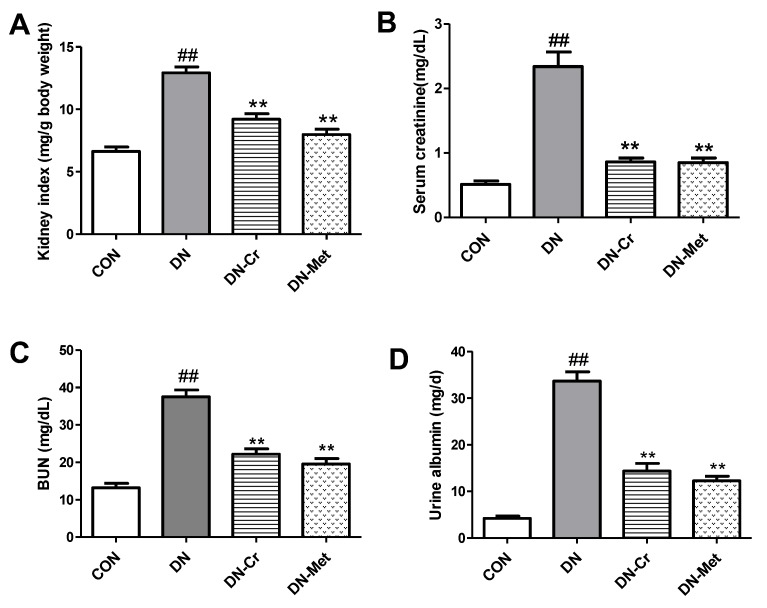
The kidney index, serum creatinine, blood urea nitrogen (BUN), and urinary albumin in each experimental group. (**A**) The kidney index in each experimental group; (**B**) The serum creatinine in each experimental group; (**C**) The blood urea nitrogen (BUN) in each experimental group; (**D**) The urinary albumin in each experimental group; CON. control group; DN. diabetic nephropathy group; DN-Cr. diabetic nephropathy rats treated with CrPic (10 mg kg^−1^ d^−1^); DN-Met. diabetic nephropathy rats treated with metformin (150 mg kg^−1^ d^−1^). Compared to the control group, ## *P* < 0.01; Compared to the DN group, ** *P* < 0.01.

As shown in Figure 3A, the kidney index of DN rats was significantly higher than that of the control (*P* < 0.01). After eight weeks of CrPic or metformin administration, the kidney index decreased, and there were significant differences in kidney index between the CrPic- or metformin-treated group and the DN group (DN-Cr vs. DN, *P* < 0.01; DN-Met vs. DN, *P* < 0.01), which indicates that CrPic can decrease the kidney index in DN rats.

Figure 3B, C, and D indicate that serum creatinine, blood urea nitrogen (BUN), as well as urinary albumin were significantly higher in DN rats compared to the control (*P* < 0.01); this was lowered by CrPic treatment, and there were significant differences in serum creatinine, BUN, and urinary albumin between the CrPic-treated group and the DN group (serum creatinine, DN-Cr vs. DN, *P*< 0.01; BUN, DN-Cr vs. DN, *P* < 0.01; urinary albumin, DN-Cr vs. DN, *P* < 0.01).

### 3.4. CrPic Inhibits Renal Oxidative Stress in Diabetic Nephropathy Rats

**Figure 4:**
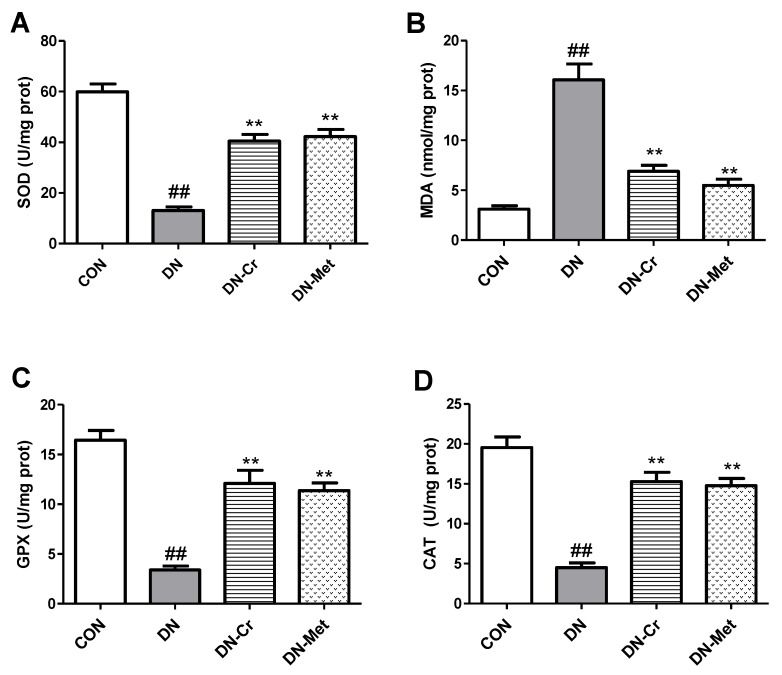
Renal MDA, SOD, GPX, and CAT levels in each experimental group. (**A**) Renal SOD levels in each experimental group; (**B**) Renal MDA levels in each experimental group; (**C**) Renal GPX levels in each experimental group; (**D**) Renal CAT levels in each experimental group; CON. control group; DN. diabetic nephropathy group; DN-Cr. diabetic nephropathy rats treated with CrPic (10 mg kg^−1^ d^−1^); DN-Met. diabetic nephropathy rats treated with metformin (150 mg kg^−1^ d^−1^). Compared to the control group, ## *P* < 0.01; Compared to the DN group, ** *P* < 0.01.

As shown in Figure 4, the concentration of malondialdehyde (MDA) in kidney homogenate was significantly higher in the DN group compared to the control (*P* < 0.01). After eight weeks of CrPic treatment, the MDA level in the DN-Cr group decreased, and the differences were found to be significant (DN-Cr vs. DN, *P* < 0.01). The biological activity of superoxide dismutase (SOD), glutathione peroxidase (GPX), and catalase (CAT) in kidney homogenate decreased significantly in the DN group, compared to the control (*P* < 0.01), and CrPic increased SOD, GPX, and CAT activity levels. There were significant differences between the antioxidant stress parameters of the CrPic-treated group and the DN group (SOD, DN-Cr vs. DN, *P* < 0.01; GPX, DN-Cr vs. DN, *P* < 0.01; CAT, DN-Cr vs. DN, *P* < 0.01).

### 3.5. CrPic Improves Renal Tissue Pathological Changes in Diabetic Nephropathy Rats

**Figure 5:**
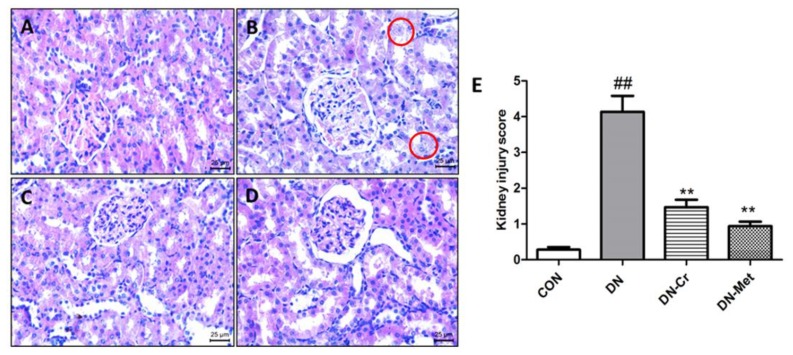
Renal tissue structure and pathological score of renal lesions in each group. H&E staining; Magnification: 400×. (**A**) The kidney of a rat in the control group; (**B**) The kidney of a rat in the DN group; (**C**) The kidney of a rat in the DN- Cr group; (**D**) The kidney of a rat in the DN-Met group; (**E**) The pathological score of renal lesions in each group. Red circles are inflammatory cells; Compared to the control group, ## *P* < 0.01; Compared to the DN group, ** *P* < 0.01.

As shown in Figure 5, there were pathological changes in the kidneys of the DN group (Figure 5B): the glomerular structure was severely damaged, the renal interstitium was widened, the capillary basement membrane was thickened, vacuolar degeneration and necrosis of the renal tubular epithelial cells were observed, and inflammatory cells were found to have increased in the renal interstitium. Compared to the DN group, the kidney structure of the CrPic treatment groups (Figure 5C), and metformin-treated group was found to be improved (Figure 5D): the glomerular volume was normal, the renal tubular endothelium was intact and smooth, the epithelial cells were arranged intact, and there was only a small amount of inflammatory cell infiltration in the interstitium. As Figure 5E indicates, the kidney injury score in the CrPic-treated group significantly decreased, when compared to the DN group (DN- Cr vs. DN, *P* < 0.01). All of the above indicate that CrPic can reverse renal tissue pathological changes in DN rats. 

### 3.6. CrPic Inhibits Renal Interstitial Fibrosis in Diabetic Nephropathy Rats

**Figure 6:**
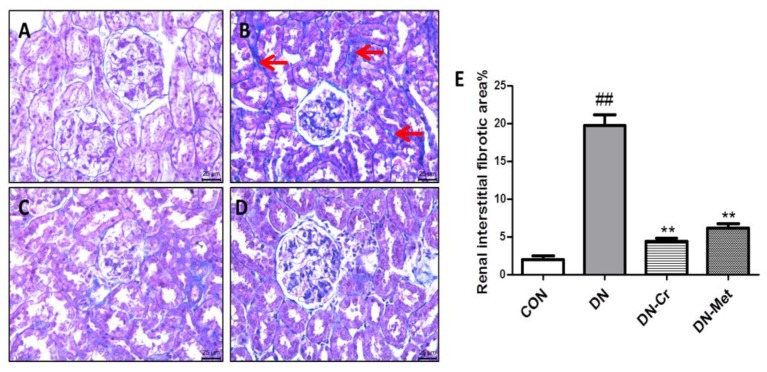
Renal interstitial fibrosis and renal interstitial fibrosis area (%) in each group. Masson’s trichrome staining; Magnification: 400×. (**A**) The kidney of a rat in the control group; (**B**) The kidney of a rat in the DN group; (**C**) The kidney of a rat in the DN- Cr group; (**D**) The kidney of a rat in the DN-Met group; (**E**) Renal interstitial fibrosis area (%) in each group. The red arrows point to collagen fibers. Compared to the control group, ## *P*< 0.01; Compared to the DN group, ** *P* < 0.01.

The kidney slides were stained with Masson’s trichrome to observe renal interstitial fibrosis. As shown in Figure 6B, there was severe renal interstitial fibrosis in the DN rats and a large distribution of collagen fibers. The degree of renal interstitial fibrosis in the CrPic-treated group was significantly lesser than that in the DN group. As indicated in Figure 6E, compared with the DN group, the percentage of renal interstitial fibrosis in the CrPic- or metformin-treated group decreased significantly (DN-Cr vs. DN, *P* < 0.01; DN-Met vs. DN, *P* < 0.01). All of this indicates that CrPic can inhibit renal interstitial fibrosis in diabetic rats. 

### 3.7. CrPic Inhibits Glomerulosclerosis in Diabetic Nephropathy Rats 

**Figure 7:**
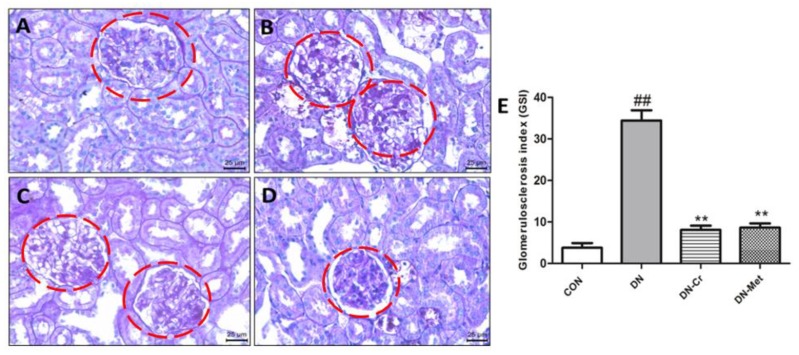
Glomerulosclerosis and glomerulosclerosis index (GSI) in each group. PAS staining; Magnification: 400×. (**A**) The kidney of a rat in the control group; (**B**) The kidney of a rat in the DN group; (**C**) The kidney of a rat in the DN-Cr group; (**D**) The kidney of a rat in the DN-Met group; (**E**) The glomerulosclerosis index (GSI) in each group. The red circles are the glomeruli; Compared to the control group, ## *P* < 0.01; Compared to the DN group, ** *P* < 0.01.

The kidney sections were stained with Periodic Acid-Schiff (PAS) to observe glomerulosclerosis. As shown in Figure 7B, in the kidney of a DN rat, there were large amounts of glycoprotein in the glomerular matrix and under the glomerular capillary endothelium. After eight weeks of CrPic treatment, glycoprotein obviously decreased in the DN-Cr (Figure 7C) and metformin-treated groups (Figure 7D).

As shown in Figure 7E, compared to the control, the glomerulosclerosis index (GSI) significantly increased in the DN group (*P* < 0.01). After eight weeks of CrPic treatment, the GSI decreased, and there were significant differences in the GSI of the DN-Cr or DN-Met group and the DN group (*P* < 0.01), thus indicating that CrPic can inhibit glomerulosclerosis in DN rats. 

### 3.8. CrPic Regulates the Renal TGF-β1/Smad Signal Pathway in Diabetic Nephropathy Rats 

**Figure 8:**
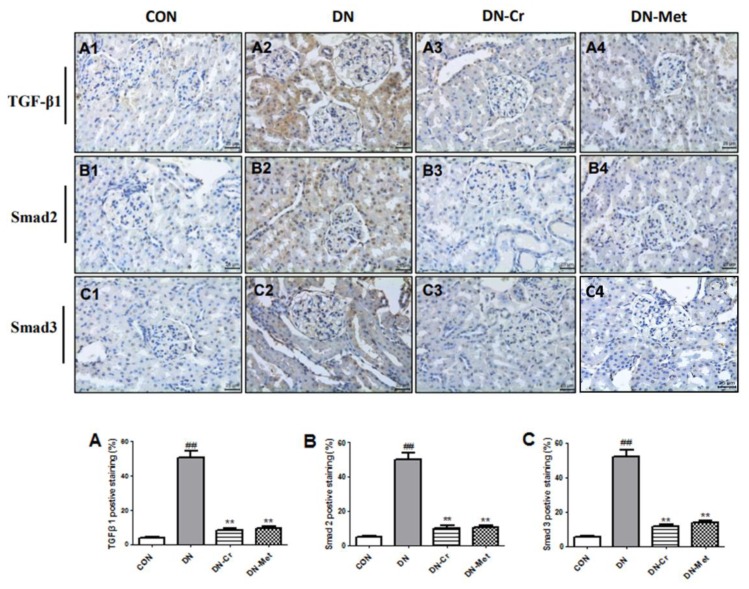
TGF-β1, Smad 2, and Smad 3 protein expression in the kidney tissues of each group. Immunohistochemical staining; Magnification: 400×. (A1–A4) TGF-β1 in the kidney of rats in each group; (B1-B4) Smad 2 in the kidney of rats in each group; (C1–C4) Smad 3 in the kidney of rats in each group; (**A**) TGF-β1 positive staining area in each group; (**B**) Smad 2 positive staining area in each group; (**C**) Smad 3 positive staining area in each group; Compared to the control group, ## *P* < 0.01; Compared to the DN group, ** *P* < 0.01.

As shown in Figure 8, when compared to the control, TGF-β1, Smad 2, and Smad 3 expression levels increased significantly in the kidney of the DN group rats (*P* < 0.01). After eight weeks of CrPic or metformin treatment, TGF-β1, Smad 2, and Smad 3 expression levels decreased, compared to the DN group. As Figure 8A–C shows, the TGF-β1, Smad 2, and Smad 3 positive staining area significantly increased in the DN group, compared with the control (*P* < 0.01). Eight weeks of CrPic or metformin treatment decreased TGF-β1, Smad 2, and Smad 3 expression levels in diabetic rats, and the positive staining area of TGF-β1, Smad 2, and Smad 3 significantly decreased in the CrPic- or metformin-treated group, as compared to the DN group (*P* < 0.01).

## 4. Discussion

Diabetic nephropathy is a serious complication in diabetic patients and the main cause of end-stage renal disease (ESRD). Therefore, the prevention and treatment of diabetic nephropathy is of great significance. CrPic is known to have a beneficial effect on diabetic rats; however, whether CrPic is beneficial for diabetic nephropathy or not has not been studied. In this study, the protective effect of CrPic on STZ-induced renal injury in diabetic rats was evaluated by its antifibrotic and antioxidative characteristics, and ability to regulate the TGF-β1/Smad signaling pathway. Our results indicate that eight weeks of CrPic supplementation can prevent diabetic nephropathy in SD rats.

The most common way to establish an animal model of diabetic nephropathy is by using streptozotocin (STZ) [15], which is characterized by high efficiency, simplicity, and stability. STZ can induce pancreatic cell apoptosis, Type 1 diabetes, diabetic nephropathy, as well as cause the proliferation of glomerular endothelial cells [16]. In this study, SD rats were given an intraperitoneal injection of streptozotocin (45 mg kg^-1^); three days after the injection, a significant increase in blood glucose, feed and water intake, and urine volume was noted. The non-fasting blood glucose level of the STZ-injected rats was found to be above 16.7 mmol/L, with positive urine protein. Renal tissue section staining showed glomerular hypertrophy, increased mesangial cells, vacuolar degeneration of renal tubular epithelial cells, and increased renal interstitial inflammatory cells, which was consistent with characteristics of early diabetic nephropathy. This indicated the successful construction of a diabetic nephropathy model.

This study revealed that CrPic has a good hypoglycemic effect and can improve symptoms of Type 1 diabetes, such as weight loss, polydipsia, and polyphagia. After eight weeks of CrPic administration, blood glucose and serum insulin levels in diabetic rats decreased, body weight increased, and water and food consumption decreased. This indicates that CrPic has a therapeutic effect in diabetics. Body weight, food or water intake, blood glucose, and insulin levels are key clinical indicators of diabetes. Although several reports discuss CrPic’s effects on body weight, blood glucose and insulin levels in diabetes [17,18,19,20], more investigation is required to better evaluate its anti-DN properties.

Serum creatinine, blood urea nitrogen (BUN), and urinary albumin are important indicators of renal function [21]. Impaired glomerular filtration membrane barrier function in patients with diabetic nephropathy often leads to increased excretion of albumin in urine, increased serum creatinine, and blood urea nitrogen [22]. Therefore, when these levels significantly increase in diabetic patients, it indicates impaired renal function. This study reveals that after eight weeks of CrPic administration, serum creatinine, BUN, and urinary albumin all significantly decreased in the CrPic-treated group, compared to the DN group, thus indicating that CrPic has a protective effect on renal function in diabetic nephropathy rats.

The “gold standard” for diabetic nephropathy diagnosis is renal pathological change [23]. The pathological characteristics of diabetic nephropathy are thickening of the glomerular basement membrane, extracellular matrix accumulation, renal interstitial inflammatory cell infiltration, renal interstitial fibrosis, and glomerular sclerosis [24,25,26]. In this study, the kidney structure of the DN rats was found to be disordered, the glomerular structure severely damaged, the capillary basement membrane thickened, and the renal tubular epithelial cells showed vacuolar degeneration; inflammatory cells were also found increased in the interstitium of the renal tubule. After eight weeks of CrPic treatment, the kidney structure was repaired, and the renal interstitial fibrosis and glomerular sclerosis were effectively alleviated, indicating that CrPic can alter renal tissue pathological change in DN rats.

Oxidative stress is a significant pathogenesis of DN, as oxidative phosphorylation of mitochondria in the kidney produces large amounts of reactive oxygen species (ROS), which ultimately affect kidney structure and physiological function [27,28]. Recent studies have confirmed that hyperglycemia-induced oxidative stress is closely related to mesangial cell and podocyte injuries, as well as interstitial fibrosis [29]. Rodent experiments have shown that antioxidant therapies can inhibit the occurrence of DN, and herbal extracts with antioxidant effects can inhibit glomerular hypertrophy and improve renal function [30]. MDA is a final decomposition product of lipid peroxidation; thus, the determination of renal MDA content can reflect the degree of renal cell damage. SOD, CAT, and GPX are important components in the body’s antioxidant defense system [31]. Our results confirm that the MDA level increased; and GPX, CAT, and SOD activities decreased in the kidney of diabetic rats, suggesting the impairment of renal redox homeostasis, accumulation of reactive oxygen species (ROS), and formation of lipid peroxidation in diabetic rats. After eight weeks of CrPic treatment, the level of MDA was normalized; and SOD, CAT, and GPX activities returned to normal. This suggests that antioxidative stress is a mechanism employed by CrPic against diabetic nephropathy.

The TGF-β1/Smads signaling pathway plays an important role in DN pathogenesis, which includes podocyte injury, basement membrane thickening, mesangial cell proliferation, renal cell apoptosis, and epithelial cell interstitial transformation, among others [32,33]. Studies have confirmed that abnormal activation of the TGF-β1/Smad signaling pathway may be the main cause of renal fibrosis in diabetic nephropathy patients [34]. Therefore, the TGF-β1/Smads signaling pathway is the theoretical basis for research on the development of functional foods or drugs that work against diabetic nephropathy. TGF-β1 is a multifunctional cytokine that has been identified as a critical regulator of ECM protein synthesis in DN, which is mainly responsible for ECM accumulation and closely related to the development of kidney fibrosis and glomerular sclerosis [35]. TGF-β1 exerts biological activities through phosphorylation of Smad2 and Smad3, which are two critical downstream mediators [36]. Several studies have demonstrated that TGF-β1/Smad2/3 signaling is highly activated in diabetic nephropathy kidneys. Thus, inhibition of the TGF-β1/Smad2/3 pathway may be a potential aim of DN therapy [37]. The immunohistochemical results in this study also showed that TGF-β1 and Smad2 or 3 expression levels were elevated in DN rats. In contrast, treatment with CrPic reversed these changes, highlighting the fact that CrPic prevents diabetes-induced kidney fibrosis and glomerular sclerosis by inhibiting the TGF-β1/Smad2/3 pathway. 

In summary, eight weeks of CrPic administration demonstrated vital renal-protective effects in diabetes-induced nephropathy; thus, it has great potential in preventing and treating diabetic nephropathy.

## 5. Conclusions

Eight weeks of CrPic supplementation was found to repair renal function and reverse renal pathological changes (renal interstitial fibrosis and glomerular sclerosis) in diabetic nephropathy rats by an antioxidative stress mechanism, as well as by inhibiting TGF-β1 and Smad2/3 expressions. CrPic could, thus, be used as a nutrient to prevent diabetic nephropathy.
